# A Systematic Review of Research into the Impact of Loose Parts Play on Children’s Cognitive, Social and Emotional Development

**DOI:** 10.1007/s12310-017-9220-9

**Published:** 2017-07-31

**Authors:** Jenny Louise Gibson, Megan Cornell, Tim Gill

**Affiliations:** 1 Centre for Research on Play in Education, Development and Learning, Faculty of Education, University of Cambridge, Cambridge, UK; 2 Independent Researcher, 58 Upper Walthamstow Road, London, E17 3QQ UK

**Keywords:** Well-being, Recess, Play, Social-emotional development, Loose parts play, Playtime

## Abstract

Loose parts play (LPP) interventions introduce moveable materials and equipment to children’s play spaces to facilitate unstructured, child-led play. Meta-analysis of previous school-based research has shown significant benefits of LPP for physical activity. In the current paper, we review the scope and quality of the quantitative evidence relating to cognitive, social and emotional outcomes. We conducted a systematic search of the literature on LPP interventions for primary school-aged children which used quantitative outcome indicators for cognitive, social and/or emotional development. Studies were screened for inclusion by two independent researchers and reviewed for content, relevant outcomes and quality indicators. Five studies met the review inclusion criteria. Two studies used a randomised controlled trial design, two studies used quasi-experimental design, and one used an observational design. Outcomes measured focused mainly on social development. With the exception of enjoyment, school satisfaction and self-esteem, emotional outcomes were almost entirely absent. No measures of cognitive or academic outcomes were found. For the studies using control groups, few differences between groups were reported, although one study found increased happiness at school and increased odds of reporting being pushed/shoved at playtime associated with intervention. Null results were found for peer acceptance, relational bullying, social competence, social skills, peer group size and psychosocial quality of life. In the non-controlled study, there were observed increases in co-operative play. There is insufficient high-quality, quantitative, empirical evidence available to determine whether or not LPP interventions have an impact on children’s cognitive, social and emotional development. We conclude our review with some recommendations which we hope will assist future research in this promising field.

## Introduction

In many people’s minds, school playtime (or ‘recess’) is the time of the school day when children take a break from learning. However, research has found that children require more sophisticated skills to engage on the playground than those required in other school contexts (Baines & Blatchford, 2010). Playtime has also been associated with the opportunity to develop friendships, which are in turn related to children’s sense of social identity and well-being (Baines, Blatchford, & Pellegrini, 2001; Gibson, Hussain, Holsgrove, Adams, & Green, 2011). Research has also linked school playtime closely to school adjustment and classroom behaviour (Pellegrini & Bohn, 2005), indicating the developmental importance of a balance between different types of learning opportunities during the school day (Jarrett et al., 1998; Pellegrini & Davis, 1993).

One significant distinction between the learning and development opportunities afforded by the playground and those afforded by the classroom is the proportion of time spent in unstructured, child-led activity. Unstructured play allows children space to choose and create their own playful activities, to navigate their social worlds, to make independent decisions and to experience the consequences of their own actions. Unstructured play is therefore thought to be a crucial context for the development of independence and emotional/behavioural self-regulation (Pellis & Pellis, 2007). In turn, self-regulatory skills have been associated with improved child well-being and academic achievement (Eisenberg, Valiente, & Eggum, 2010; Zimmerman, 1990). Children may engage in various types of play on the playground (including social co-operation, pretence, rough and tumble, games with rules, and more) all of which are significant in their own right and thought to be of developmental significance.

Given the potential developmental significance of unstructured play, it is important that schools provide high-quality opportunities for children to engage in it. One of the features of outdoor playtime which most supports independent development is the relative lack of adult supervision compared to that found in classroom contexts (Blatchford, 1989). Therefore, interventions designed to capitalise on the inherent developmental opportunities of playtime may be most effective when they can preserve the child-led focus, rather than, say, introducing adult-led sports activities.

The quality of the play environment has also been found to be influential in the enjoyment and benefits that children get from playtime, as well as the different types of play in which they engage (Powell, 2007; White, 2013). Importantly, qualitative research has demonstrated that the features valued by children in the playground environment may not match the assumptions of adults. Factor noted that seemingly incidental physical features of playground such as drain covers can become a focal point for play (Factor, 2004) while playground markings such as painted lines or spots on the floor, often implemented by well-meaning adults as a means of enhancing play space, may have little effect on children’s play behaviours (Cardon, Labarque, Smits, & Bourdeaudhuij, 2009; Stratton, 2000). Evidence regarding engagement with fixed play equipment is mixed with some research showing a decrease in physical activity associated with fixed items and others an increase physical activity (Dyment & O’Connell, 2013; Frost, 1990; Gubbels, Van Kann, & Jansen, 2012). However, our primary interest in the present study is on the provision of loose parts materials at playtime.

Loose parts play (LPP) is a technique that has been developed as a means improving the quality of the ‘play offer’ while maximising the opportunities for child-led play and opportunities for engagement. Typically, this involves the introduction of moveable materials and equipment to children’s play spaces and inviting them to engage as they wish with little or no adult direction. The introduction of loose parts with the intention of enhancing engagement has its roots in the principles of the ‘Theory of Loose Parts’ expounded by Nicholson (1972). Nicholson proposed that,

*in any environment, both the degree of inventiveness and creativity, and the possibility of discovery, are directly proportional to the number and kind of variables in it.* -*Nicholson,* 1972 *, p. 6.*

Nicholson’s ideas were developed in the context of design theory and how an individual’s environment could be designed to optimise creativity and engagement. Applied to the context of the school playground, the idea is to introduce moveable materials, (e.g. old milk crates, nets, tyres) to play spaces so that children can take advantage of the opportunities for creativity and discovery afforded by them (Bundy et al., 2011). The current commonly implemented models of LPP in educational settings (and beyond) have emerged from playwork practice and have been developed with the principles of every child’s right to play and the importance of child-led engagement in play as underpinning values (Fjortoft & Sageie, 2000; Maxwell, Mitchell, & Evans, 2008).

Quantitatively oriented outcomes for playtime interventions have been studied mostly with respect to physical activity (PA), and there is a growing and robust evidence base in this area (Dobbins, Husson, DeCorby, & LaRocca, 2013; Engelen et al., 2013; Houser, Roach, Stone, Turner, & Kirk, 2016; Ridgers, Carter, Stratton, & McKenzie, 2011). With respect to LPP interventions specifically, in a cluster-randomised trial of an LPP + parent education intervention, Engelen et al. (2013) used accelerometer-based measures and found significant increases in step counts and minutes spent in moderate-vigorous physical activity (MPVA), as well as a decrease in sedentary behaviour in the intervention condition when compared to controls. Similar effects in favour of the intervention group were found in a quasi-experimental study carried out by Hyndman and colleagues, which found significant increases in steps per minute.

Outcomes in domains other than PA, such as cognitive, social and emotional development have received less attention in studies using quantitative approaches; however, qualitative studies have reported encouraging results (James, 2012; Lester, Jones, & Russell, 2010). Based on interviews with participants from schools implementing an intervention that included (but was not limited to) LPP, Lester, Jones, and Russell (2010) reported benefits including improved social behaviour and academic engagement. James (2012) also used interview methods to evaluate the impact of LPP and participants reported improvements in self-esteem, confidence, social inclusion and happiness, alongside reductions in boredom and aggression associated with playtime.

These reported outcomes, which we here broadly summarise as ‘cognitive, social and emotional,’ are of great interest to practitioners in education, given increased recognition of the importance of educational environments that promote both well-being and learning. A full analysis of the extensive literature on the potential mechanisms behind effects of unstructured play on children’s cognitive, social and emotional development is beyond the scope of this introduction; however, we discuss here some possible hypotheses linking to LPP interventions to these outcomes before going on to describe the aims and scope of our review.

Firstly, the opportunity for risk-taking in play has been linked to positive developmental outcomes (Gill, 2007; Lavrysen, Bertrands, Leyssen, Smets, Vanderspikken, & De Graef, 2017). It is thought that the opportunity for risk-taking improves children’s competencies in risk management and risk perception. In addition, social skills may be enhanced through opportunities for collaboration with older peers, as children collectively decide and learn how to manage risk. Although Bundy and colleagues (Bundy et al., 2009) have done excellent qualitative work on the perception of risk, to the best of our knowledge, no studies have attempted to quantitatively measure risk-taking in LPP and to link it to theoretically associated outcomes.

The power of shared resources is also a potential mechanism via which LPP interventions may influence socio-emotional development. Spinrad and colleagues found solitary and reticent play behaviour to be associated with peer exclusion, anxiety issues and poor emotional regulation (Spinrad et al., 2004). It therefore seems reasonable to hypothesise that shared resources that facilitate collaborative play may improve outcomes associated with emotional regulation.

An indirect route for improvement in cognitive, social and emotional outcomes could be through the influence of physical activity (PA). As discussed above, LPP interventions have been consistently associated with increased PA. Research has linked physical activity not only to physical health but also to mental well-being (Ahn & Fedewa, 2011) and academic achievement (Singh, 2012). It is possible therefore that PA represents a mediating variable between increased engagement in play and cognitive, social and emotional outcomes.

It is also likely that intrinsic motivation and freedom to enjoy the challenges of play for its own sake have a role to play. For some children, the inherent social demands of the playground can seem daunting and it may be the case that engagement with objects in the playground provides the optimal balance between social challenge and social competence—producing a play state akin to ‘flow’ (Nakamura & Csikszentmihalyi, 2014). This idea is considerably more speculative than the ones discussed above. However, it does suggest some testable hypotheses; e.g. enjoyment of play may increase with increasing challenges up to a maximum point after which enjoyment may decline as challenges become too great.

In summary, a number of theoretical accounts have linked play behaviours in unstructured contexts to improved cognitive, social and emotional outcomes for children. Moreover, qualitative studies have suggested LPP represents a good way for schools to foster this type of play and improve these outcomes for children. Based on this information, we wished to investigate the scope, quantity and quality of quantitative evidence of the effects of LPP interventions on social, emotional and cognitive development. Our decision to investigate this area was also informed by the involvement of non-academic partners in our research group discussions. Stakeholders reported increasing uptake of LPP interventions in local schools and wished to know more about the associated evidence base to help inform decision making.

### Review Aims and Objectives

Following Gough, Oliver and Thomas’ recommendation that systematic reviews (Gough, Oliver, & Thomas, 2012) should answer the questions;

“*what is already known and how do we know it?*” and, if necessary, “*what more do we need to know and how can we know it?*” (Gough et al., 2012, p. 3)

we developed the following research question to guide our review:

1. *What are the effects of LPP interventions on cognitive, social and emotional outcomes in primary school-aged children?*

Additionally, we aimed to address some broader questions in the field that had emerged from the stakeholder discussions;

2. *How have LPP interventions have been studied in quantitatively in relation to cognitive, social and emotional development, including information about types of study designs and outcome measures?*
3. *What considerations should inform future studies of LPP interventions?*

## Method

Methods for the study were agreed by the research team in consultation with stakeholders in advance of the review. Copies of the protocol and data extraction table are available from the corresponding author. The review was not pre-registered.

### Study Eligibility

The criteria for including studies in our review were determined by the aims of our research as set out in the introduction, as well as by some pragmatic considerations. The inclusion criteria are as follows:

The paper or report should be:

- a study or evaluation of the introduction of loose materials (e.g. scrap items, construction materials, sports equipment) into school playgrounds for children to use freely during breaktimes.
- related to primary school-aged children (4–12 years).
- concerned with primarily quantitative outcome indicators (questionnaires, psychological tests, observational sampling), or using mixed methods where at least one quantitative measure focussed on non-PA outcomes.
- concerned with outcomes not solely related to physical activity (PA).
- a study or evaluation carried out or commissioned by academic institutions or authoritative agencies (established NGOs, think tanks, national governments).
- written in English.
- published between 01/01/2000 and 01/06/2017.

Although discussions with our stakeholder group identified that the primary outcomes of interest were cognitive, social and emotional, we did not constrain study eligibility on this basis. These constructs are extremely broad, and this raised the possibility of introducing a high degree of unwanted inter-assessor variability if we introduced limits early in the study selection process. Additionally, we developed a set of exclusion criteria in anticipation of possible ambiguities. The exclusion criteria are as follows:

The paper or report should *not* be:

- related to structured programmes such as sports-based, arts-based or lesson-based programmes that are adult led and directed.
- entirely devoted to reporting PA outcomes.
- entirely qualitative (e.g. interviews or focus groups only).

Note that although we did not systematically search the grey literature (see below), we did not have exclusionary criteria based on this factor. Thus, we considered for inclusion any studies that came to our attention regardless of their peer-review or publication status.

### Search Strategy

A combination of electronic- and hand-searching was used to identify studies. After consultation with an academic librarian, the following electronic databases were selected for our search:

- British Education Index
- Child Development & Adolescent Studies
- ERIC
- PsychInfo
- Science Direct
- Scopus
- Web of Science

Papers were also sought by reading through bibliographies of studies and reports already known to the search team, and by contacting researchers in the field to ask whether they knew of any relevant material. Papers or reports discovered in this way were evaluated in the same way as papers retrieved from our electronic searches. We did not conduct a direct search of the ‘grey literature’ as a thorough search of this material was beyond the resources of the current project.

To refine our search terms, a number of initial scoping searches were carried out. Results from the scoping searches were scanned for relevance, and terms that consistently yielded false positives (i.e. irrelevant results) were excluded. Relevant articles were used to identify additional key words. As a result of this process, the terms #block play and #moveable parts were excluded from the search, while the terms #playthings, #outdoor play and #play materials were added. The final list of search terms is as follows:

*Materials Synonyms* #Loose materials OR #Loose parts OR #Modular play OR #Scrap materials OR #Playpods OR #Play materials #Playthings

AND

*Location Synonyms* #Breaktime OR #Free play OR #Lunchtime OR #Play OR #Playground OR #Playtime OR #Primary school OR #Recess OR #School OR #Schoolyard OR #Outdoor play

All final searches were constrained to date of publication between 01/1990 and 06/2017. The first searches were carried out in November 2016 and updated in February and then June 2017. Where databases had an option to constrain by subject we added limits to constrain findings to behavioural/psychological sciences, education, neuroscience, social sciences, humanities and medicine only. This proved very useful in limiting the number of hits in disciplines related to Engineering as they are interested in the term Loose Parts for very different reasons to our own! Finally, we set parameters to include studies published in English only, as this was the main language of the research team members.

Searches using the terms listed above were run in each of the selected electronic databases, and results were imported into the electronic reference management software Zotero. Zotero’s dedupe function was used to assist identification and deletion of duplicate hits.

Studies then were screened using a 2-step screening method. Firstly, the first author sifted the search results, using study title to exclude obviously irrelevant studies. Examples of studies excluded at this stage include studies of human sexual behaviour, and animal studies. For the next screening step, the first and second authors independently read all study abstracts in order to determine which studies could be immediately excluded with reference to the inclusion/exclusion criteria above. Where disagreements were identified, the inclusion/exclusion criteria were used as the basis for discussion between raters and a decision was then reached jointly.

Following the screening stage, full copies of the remaining papers were obtained and the first and second authors independently read each one, noting whether or not the studies met the review inclusion criteria. When studies were excluded at this stage, a short note was added to the electronic records, noting the reason for exclusion. Percentage agreement between reviewers was calculated. Reviewers’ notes in conjunction with the inclusion/exclusion criteria were used as the basis for resolving disagreement.

For the final set of studies, an excel spreadsheet was set up to record the study characteristics and findings. A copy of the spreadsheet is available from the corresponding author.

### Planned Analyses

Finding an answer to the research question, *what are the effects of LPP* *interventions on cognitive, social and emotional outcomes in primary school-aged children?* involved finding studies which had addressed these issues. Given our existing knowledge of the field, we judged it unlikely that we would find substantive numbers of studies and therefore we did not plan a priori to carry out a statistical meta-analysis as part of the current review. Instead we planned to use a narrative, thematic approach to synthesising the relevant information including study design, types of outcome measures used, population sampled, hypothesised mechanisms of effect and so on.

In order to assess the quality of the research relating to the risk of bias in relation to RQ1, we used the Canadian Effective Public Health Practice Project (EPHPP) method of appraisal to consider bias/quality issues arising from the following: study design, selection of participants, blinding, and data collection methods and dropout (EPHPP, n.d.). In addition, we added in quality indicators considered important by the stakeholder group including Ethical Review, Conflict of Interest (CoI) declaration, Pre-registration of the Study and Funding Source. Inter-rater reliability for quality assessment was carried out by the first author and a research assistant.

## Results

### Included Studies

**Fig. 1:**
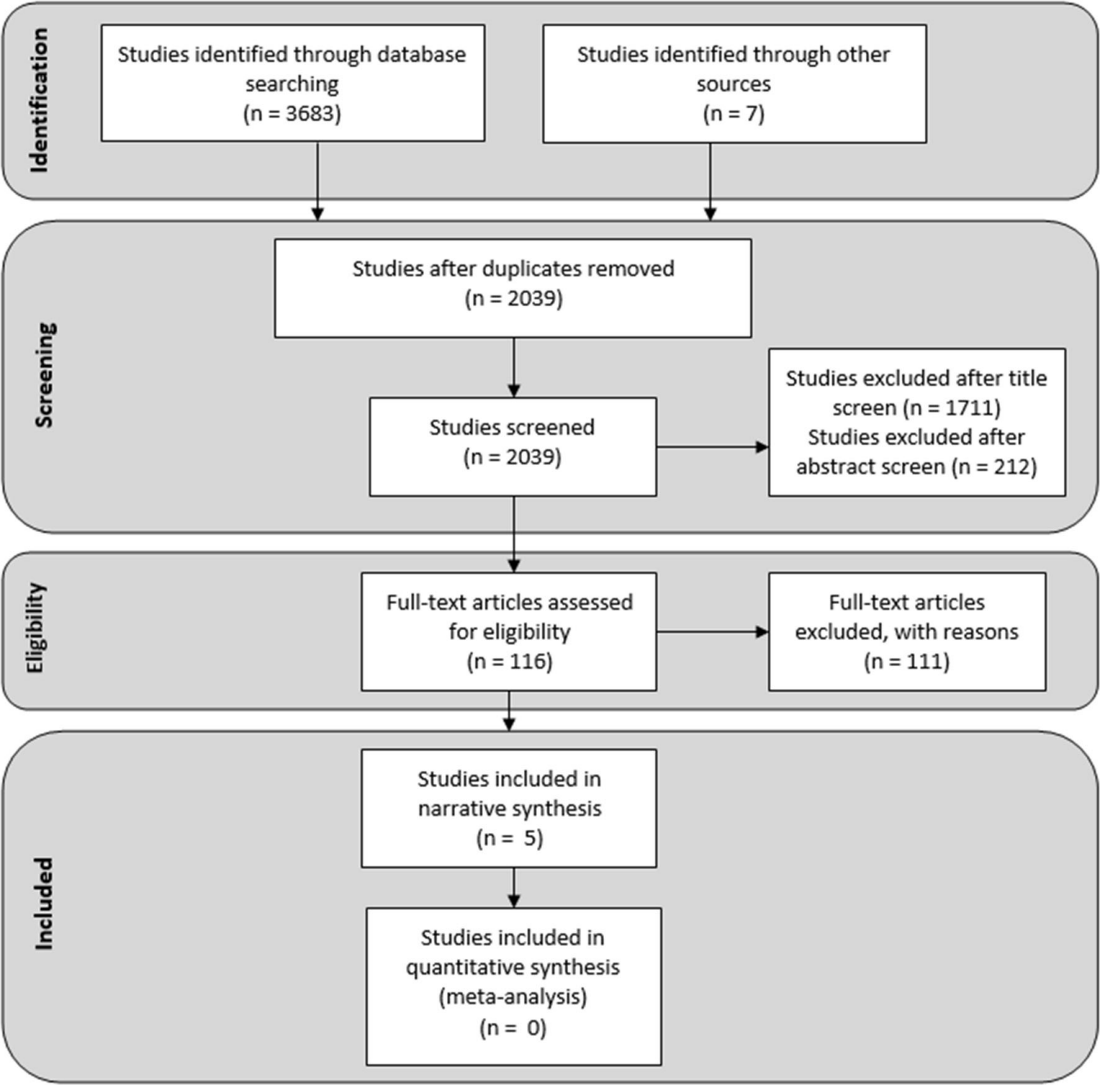
Flow diagram for study screening and inclusion. Based on Moher et al. (2009)

The number of studies at each stage of the review is reported in Fig. 1.

The five studies included in the final review are summarised in Table 1. Inter-rater reliability for study inclusion/exclusion at the ‘Eligibility’ stage of the process was *κ* = 0.92 (95% CI 0.87–0.92).

**Table 1:** Studies included in the systematic review

Study	Participants	Aims	Design	Outcome measures	Findings
Barton, Sandercock, Pretty, and Wood (2015) *International Journal of Environmental Health Research*	52 children aged 8–9 years Sample drawn from 2 UK primary schools, 1 urban and 1 rural	To compare the effects of 2 playtime interventions: (1) Provision of loose sports equipment (2) Nature-based orienteering activity	Pre- and post-intervention measurement, no control group or randomisation to condition The 2 interventions were made available in each school for 5 consecutive days each Outcome measures were taken pre- and post each intervention	Change in Rosenberg self-esteem (SE) scale 10 item self-rated questionnaire. Authors adapted for use with children	Changes in SE scores were not significantly associated with one intervention compared to another
Bundy, Wyver, Naughton, Engelen, and Tranter (2016) Unpublished/under review	226 children 5–7 years Samples drawn from 12 Australian primary schools	To explore effectiveness a loose parts play intervention on children’s PA, play, perceived competence, social acceptance and social skills	Cluster-randomised controlled trial Each of the 12 schools was randomised to intervention or control Outcome measures collected at baseline and after 13 weeks	Video observations of play, each child observed for 15 min and coded for time spent in play and number of playmates The pictorial scale of perceived competence and social acceptance for young children (PSPCSAYC) The social skills improvement system rating scale (SSIS-RS) Field notes	From video data, there was no statistically significant change in time spent engaged in play, although effect size was interpreted as a potentially clinically significant increase in engagement There were no differences found for number of playmates across conditions From the PSPCSAYC data, no changes in social competence or peer acceptance were found as a result of the intervention SSIS-RS showed no changes in social skills as a result of the intervention Field notes suggest teachers’ perceived improved behaviour and social skills, increased creativity and play
Farmer et al. (2017) *Pediatrics*	840 children aged 6–9 years Control—422 children Intervention—418 children Samples drawn from 16 New Zealand primary schools	To explore whether a playtime intervention (that included an LPP component) affected children’s interactions, especially negative interactions such as bullying	Cluster-randomised controlled trial Each of the 16 schools was randomised to intervention or control Outcome measures collected at baseline, 1- and 2-year follow-ups	Peer relations questionnaire revised (PRAQ-R). This is a questionnaire measure for multi-informants: child (10 items), parent (3 items) and teacher (4 items)	Intervention children more likely to report being happy at school and playing with lots of children at 2-year follow-up. This group were less likely to report liking their classmates No group differences were observed in verbal or relational bullying. Intervention children were more likely to report being pushed/shoved at 2 years, but were less likely to tell a teacher about it Parents reported intervention children more likely to have happy relationships at 1 year, but less likely at 2 years Teachers reported few differences between intervention and control; however more intervention teachers reported observing bullying at 1 year and exclusion at 2 years
Hyndman, Benson, Ullah, and Telford (2014b) *BMC Public Health*	279 children aged 5–12 years Control—156 children Intervention—123 children Samples drawn from 2 Australian primary schools	To evaluate the effects of the LEAP intervention on quality of life (QOL), enjoyment and participation in PA	Quasi-experimental. No randomisation to condition. Matched control group used Outcome measures collected at baseline and post-intervention (7 weeks) and follow-up (8 months)	Pediatric quality of life inventory 4.0 (QoL) 23 item, child completed questionnaire including PA and psychosocial aspects of QoL Lunchtime enjoyment of activity (LEAP) questionnaire 39 item, child completed questionnaire	At the 7-week follow-up intervention group had higher enjoyment of intra-personal play activities. This difference was not maintained at the 8-month follow-up No treatment effects on psychosocial aspects of QoL were observed at 7 weeks or 8 months
Kuh, Ponte, and Chau (2013) *Children Youth and Environments*	90 children aged 4–8 years Sample drawn from an elementary school in USA	To examine the effects of an extensive ‘playscaping’ intervention This included the introduction of loose parts, although this is not the focus of the study	Mixed methods. Observational study 30 randomly selected children observed at baseline, immediately post-intervention and 6-month post-intervention	Outdoor play inventory A time-sampling observation strategy coding play styles, play patterns and play characteristics	Complex intervention makes it difficult to isolate effects of loose parts, although importance of loose parts was a theme emerging from the qualitative work Time sampling revealed a significant difference in observed amount of co-operative behaviour between children

Four of the included studies are published in peer-reviewed journals, and one is an as yet unpublished manuscript.

### Quality Assessment

**Fig. 2:**
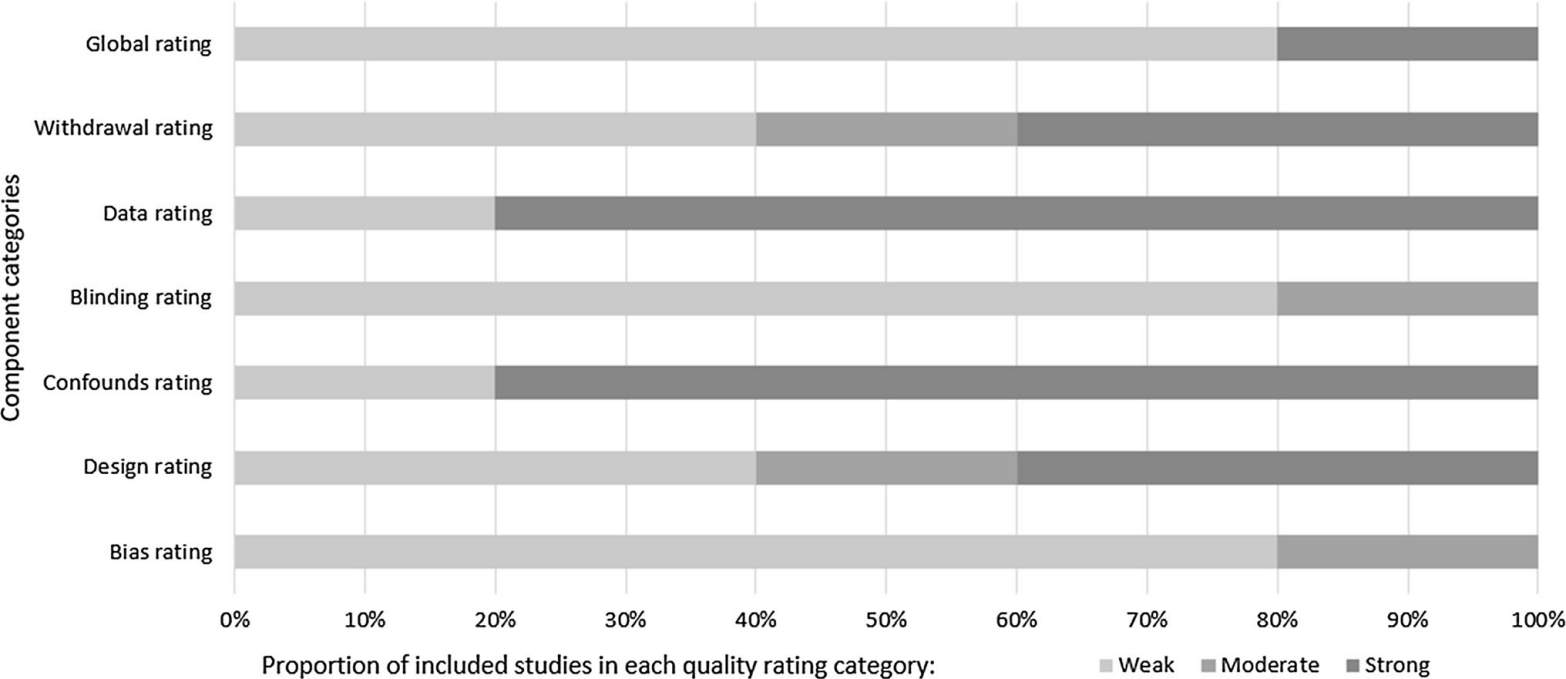
Proportion of included studies (*n* = 5) classified as *weak*, *moderate* or *strong* for each of the component rating categories of the effective public health practice project quality assessment tool

**Fig. 3:**
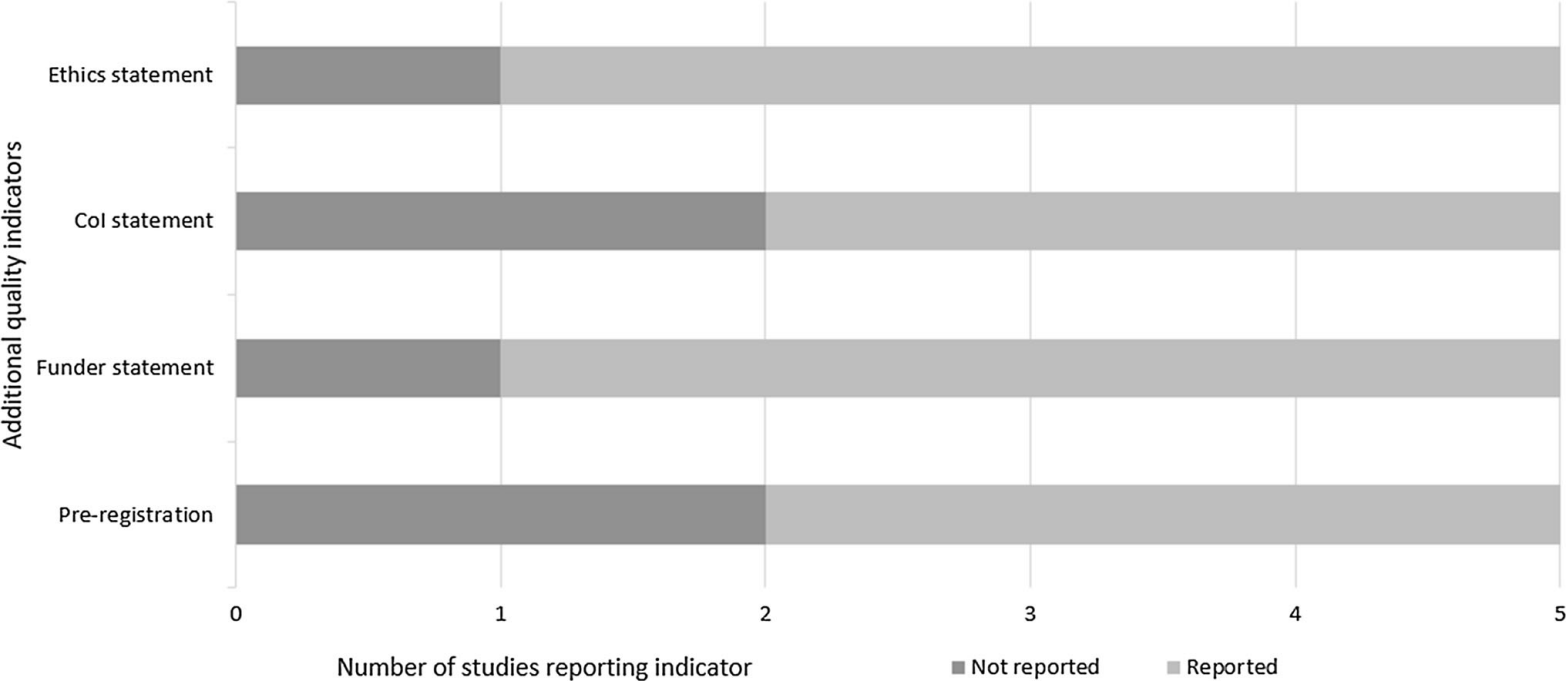
Number of studies reporting/not reporting on stakeholder agreed additional quality indicators

The quality assessments for different features of the studies in relation to our primary research question are summarised in Figs. 2 and 3. Figure 2 shows quality ratings by category from the EPHPP quality assessment tool.

Inter-rater reliability for quality assessment was *κ* = 0.69 (95% CI 0.45–0.93). Differences in ratings arose from differing interpretations of the studies (as opposed to different interpretation of the rating criteria), and most frequently occurred in the ‘bias’ rating category. Differences were resolved by discussion and with reference to the online documentation for EPHPP.

Figure 3 shows the number of studies reporting additional features considered important for quality—ethical procedures, conflict of interest (CoI) declarations, funding sources and study pre-registration.

### Narrative Synthesis

#### Study Characteristics

Two studies were conducted in Australia, one in New Zealand, one in the UK and one in the USA. All studies except one were published in peer-reviewed journals (the Bundy and colleagues study was kindly provided to us as a manuscript in preparation).

#### Interventions

Three of the included studies examine LPP interventions which introduce recycled scrap materials to the playground (Bundy, Wyver, Naughton, Engelen, & Tranter, n.d.; Farmer et al., 2017; Hyndman et al., 2014b). The duration of this type of scrap intervention ranged from 7 weeks to 1 year. In the Farmer and colleagues study, LPP was one of a number of components in an intervention package designed to improve opportunities for risky and challenging play. One study implemented more traditional loose sports materials such as skipping ropes, balls and Frisbees, over a short period of 5 consecutive days (Barton et al., 2015). Finally, the study by Kuh and colleagues evaluated LPP as part of a much larger-scale ‘playscaping’ exercise, where the whole school grounds were transformed over 3 months in the summer (Kuh et al., 2013).

#### Participant Characteristics

Across all studies, participating children were aged between 4 and 12 years old and attending mainstream schools. Ethnicity and SES data were not consistently reported by the studies making it difficult to aggregate this information.

As evident from the quality assessment above, sampling considerations are important in intervention studies. The ‘target population’ for LPP interventions was not always easy to ascertain, leading to some inter-rater disagreement in the quality ratings. The Bundy study used a random sample of schools in a fairly broad geographical area, while in contrast the Kuh study randomly sampled participants from a single school.

#### Study Designs

In terms of study design, of the five included studies, two used a cluster-randomised design, one used a quasi-experimental design, and two used observational designs. Both the study by Bundy and colleagues (2016) and the study by Farmer and colleagues (2017) adopt ‘cluster-randomised’ designs, where the random allocation of participants to intervention or control group occurred at the level of the school, rather than individual children. The Hyndman, Benson, Ullah and Telford study used a quasi-experimental design, with an intervention group and a matched control group (Hyndman et al., 2014b). The remaining two studies used observational designs (Barton et al., 2015; Kuh et al., 2013), meaning that baseline and post-intervention measurements were taken but control groups were not used.

#### Measures

No two studies shared an outcome measurement tool in common, although some methodological approaches were shared, with 3 studies using video coding of observations and 4 studies using questionnaires. All but one study investigated outcomes associated with aspects of social development, examples include co-operative play, prosocial behaviour, experience of bullying and psychosocial quality of life. Emotional outcomes were measured in 3 studies: Self-esteem in the Barton and colleagues study, Enjoyment in the Hyndman and colleagues study, and Happiness at school in the Farmer and colleagues study. No study included in the review used assessment-based indicators of cognitive or academic outcomes, although the Bundy and colleagues paper does contain ratings of self- and teacher perceived academic competence. We now describe these outcome measures in detail for each study before going on to summarise findings.

The Barton and colleagues study (Barton et al., 2015) investigated effects of the introduction of loose sports equipment on children’s physical activity (PA) and self-esteem. Self-esteem (SE) was measured at baseline and post-intervention using the 10 item, well-established, Rosenberg SE self-report questionnaire (Rosenberg, 1965). The authors report good test–retest reliability (*r*s ranging from 0.82 to 0.99) and good internal consistency (Cronbach’s alpha ranging from 0.77 to 0.88) for previous datasets although not for the sample in their study.

The paper by Hyndman and colleagues included in this synthesis (Hyndman et al., 2014b) reports on two measures which relate to outcomes other than PA (the study’s primary outcome measure). (1) The Pediatric Quality of Life Inventory 4.0 (QoL, Varni & Limbers, 2009) including a sub-scale which focuses on psychosocial development, and (2) The Lunchtime Enjoyment of Activity and Play (LEAP) Questionnaire (Hyndman, Telford, Finch, Ullah, & Benson, 2013) which aims to measure children’s enjoyment of physical, interpersonal (i.e. social) and intra-personal (i.e. individual) aspects of play. For both measures, the authors report the measures have good reliability and validity, citing a validation paper as evidence, but they do not report the co-efficients directly, or for the dataset under analysis. These measures were completed with a subset of the main sample, composed of those children aged 8–12 years. Presumably, this decision was taken due to practical difficulties in using self-report questionnaires with younger children.

The study reported by Farmer and colleagues used the well-established Peer Relations Questionnaire Revised (PRAQ-R). They used a multi-informant approach, with a different number of questions per category of respondent: child (10 items), parent (3 items) and teacher (8 items). Reliability and validity for the subset of questions adopted for the study is not reported. Outcomes were analysed on an item by item basis, rather than using scales summing across all items.

The most comprehensive set of quantitative indicators from an included study is to be found in the unpublished manuscript supplied to us by Bundy and colleagues. This study used a combination of systematic video coding, child self-report and teacher report to measure outcomes related to social and emotional development. Video recordings were taken for 15 min each day during the intervention period. An independent researcher (unaware of the study hypotheses) used the footage to note and quantify pre-specified social and play behaviours. The coding scheme used is not reported in detail; however, the authors state that the behaviours of interest were ‘categories of play and non-play, as well as quantification of social interactions’. A third of the video sample was coded for inter-rater reliability; no specific reliability co-efficient is reported, but the authors report agreement was ‘almost perfect.’

Children’s self-perceptions of their competence in physical and academic domains, together with their perceptions of social acceptance by peers and caregivers, were measured using the Harter and Pike Pictorial Scale of Perceived Competence and Social Acceptance for Young Children (PSPCSAYC) (Harter & Pike, 1984). This measure asks children to report their own assessment of their skills in these domains, using a series of pictorial prompts. The authors report ‘reliability between 0.75 and 0.89’; we assume this refers to the internal consistency of the scale in previous studies although this is not explicitly stated. Social skills were also assessed via the Social Skills Improvement System Rating Scale (SSIS-RS, Gresham & Elliott, 2008), which is a parent or teacher questionnaire used in the assessment of children’s social development. Again, good reliability and validity information are available from the cited study.

The study by Kuh et al. (2013) used a mixed methods approach, where data from systematic observations were combined with field notes and semi-structured interviews with children. Observers were trained to observe children’s behaviour live on the playground and to record the nature and duration of play activities at timed intervals of 30 s. Inter-rater reliability is reported as *κ* = 0.78, although it is not clear if this was for the observer training data or the study data. These frequency data were combined with field notes on play narratives and with comments from the children to facilitate interpretation. The pre- and post-intervention measurements were taken on a randomly picked sample each time and therefore represent changes in group behaviour, rather than changes at the individual level.

#### Findings

Having summarised some of the methodological approaches used, we now report the findings. Studies showed good awareness of potential confounding variables, and all studies included measures to control effects of at least some of the following: age, gender, SES and baseline scores. Differences in the playground environmental context were accounted for statistically in one study only, and this only accounted for space available per child. Other studies reported differences in playground type between activities, but these were either not controlled or were part of the intervention ethos itself.

Regarding social outcomes, for the studies with the most robust designs, few statistically significant intervention effects were observed. In the Bundy et al. study, null findings were reported for; engagement in play (*β* = 11.8, 95% CI −1.3 to 24.8, *p* = 0.08, *d* = 0.27), self-rated peer social competence (*β* = −0.13, 95% CI −0.29 to −0.28, *p* = 0.11) and teacher-rated social skills (*β* = −1.15 to 2.96, *p* = 0.1–0.4). For the Farmer et al. study null findings were reported as follows, Child:^1^ Liking classmates at 1 year (Odds Ratio (OR) = 0.83, 95% CI 0.64–1.07, *p* = 0.15), Liking playtime (OR = 1.06, 95% CI 0.75–1.50, *p* = 0.76), Playing with others at 2 years (OR = 1.00, 95% CI 0.59–1.70, *p* = 0.99), Liking school (OR = 0.65, 95% CI 0.41–1.03, *p* = 0.07), Verbal abuse at playtime (OR = 0.97, 95% CI 0.73–1.29, *p* = 0.82), Exclusion during playtime (OR = 1.14, 95% CI 0.69–1.90, *p* = 0.61), Being told off by a teacher (OR = 1.18, 0.80–1.75, *p* = 0.40) and Reporting bullying at 1 year (OR = 1.07, 95% CI 0.76–1.50, *p* = 0.72). Parent: Child upset by bullying at school (OR = 1.27, 95% CI 0.82–1.97, *p* = 0.29), Child has been bullied (OR = 1.60, 95% CI 0.97–2.65, *p* = 0.07). Teacher: Again mostly null findings were reported including: Frequency of reported bullying (OR = 0.01, 95% CI −0.15 to 0.16), school safety (OR = 0.12, 95% CI −0.07 to 0.30, *p* = 0.19), name-calling (OR = 0.08, 95% CI 0.13–0.29, *p* = 0.43), amongst others.

Likewise, for the quasi-experimental study no differences between intervention and control groups were found for the psychosocial QoL, nor for the interpersonal aspect of the LEAP questionnaire. In the observational study (Kuh et al., 2013), a significant increase in co-operative behaviour was observed after the implementation of the intervention.

A couple of statistically significant between-group differences in social outcomes were observed in the Farmer study: intervention children reported playing with more children at 1-year follow-up (OR = 1.66; 95% CI: 1.29–2.15), being pushed/shoved more at 2-year follow-up (OR: 1.33; 95% CI: 1.03–1.71), and less likely to tell a teacher about bullying (OR: 0.69; 95% CI: 0.52–0.92) at 2 years. Corrections for multiple comparisons were not made in the statistical analysis reported.

With reference to emotional outcomes, the pre- and post-test single group study by Barton and colleagues did not find any significant changes in self-esteem in children exposed to LPP (mean change = 1.53, SD = 5.94) compared to an orienteering activity (mean change = 1.32, SD = 4.66). Hyndman and colleagues found a small effect of LPP intervention on increased intra-personal enjoyment at the 7 weeks’ time point in their intervention (+0.24 adjusted mean change, 95% CI = 0.004–0.48, *p* = 0.045). Meanwhile, Farmer and colleagues found higher odds of children in the intervention group being happy at school at 2 years (OR = 1.64, 95% CI 1.20–2.25).

For academic outcomes, the Bundy and colleagues study reported no statistically significant changes were observed in teacher-rated academic competence (*t* = 0.13, 95% CI = −0.03 to 0.29, *p* = 0.11) and a similar outcome for self-ratings (co-efficients not reported in paper).

## Discussion

The results from the systematic review demonstrate that the amount of high-quality quantitative evidence linking LPP interventions to outcomes other than physical activity is extremely limited. The ‘gold standard’ for quantitative evaluation of intervention studies is the randomised controlled trial (or meta-analysis of several RCTs, Greenhalgh, 2014), and only two included studies met this benchmark. In the three studies that have taken quantitative approaches to measuring cognitive, social and emotional outcomes in robust study designs using control groups, there is little evidence of a sustained intervention effect in these domains. In designs without control groups, evidence is mixed, with Barton and colleagues reporting no changes in self-esteem after intervention, and Kuh & colleagues reporting increased co-operative play. Overall, the evidence from the quality assessment pictured in Fig. 2 shows that the evidence has high risk of bias and that there is limited high-quality data available.

No study included in the review used objective indicators related to cognitive or academic outcomes. As the development of cognitive-academic skills are often linked to learning via playful experiences in the literature, this is a surprising a gap in the outcomes investigated in LPP research (Berk & Meyers, 2013). Qualitative research in recent study by Hyndman and colleagues showed promising evidence of learning in the areas of Health and Physical Education, and this gives further support to the idea of relating LPP interventions to curriculum related outcomes (Hyndman, Mahony, Te Ava, Smith, & Nutton, 2016).

The mostly null findings from the review are somewhat at odds with the available qualitative evidence. For example, in the mixed-methods study by Bundy et al. included in the review (Bundy et al., 2016), field notes were used to record researchers’ experiences, thoughts and informal observations. Analysis of the notes showed that teachers reported higher levels of creativity and many also reported improvements in social play, and general behaviour. Another study by the same group using qualitative interviews with teachers found unanimous reports of increased creative play and majority reports of improved activity and reduced levels of playground aggression. Similarly in a qualitative evaluation of their LPP intervention, Hyndman and colleagues report via field notes and teacher focus group data that children showed levels of creativity, engagement, pleasure and problem solving (Hyndman, Benson, & Telford, 2014a; Hyndman et al., 2014b). Additionally, social skills such as negotiation, inclusion, team work and co-operation between children were also reported to have increased.

The divergence between the quantitative and qualitative evidence, coupled with the scarcity of robust study designs, suggests that it would be premature to use the review findings to conclude that LPP interventions do not influence children’s cognitive, social or emotional development. Note that for the evidence reported in this review, the null findings mean that no differences between the intervention and control groups were detected—not that the interventions were definitely ineffective, and there is little suggestion of negative or undesirable effects.

However, the finding of increased pushing/shoving and less reporting such behaviour to adults reported by Farmer and colleagues is noteworthy for being a potential challenge to a characterisation of LPP interventions as at best probably beneficial and at least ‘mostly harmless.’ The authors report that this finding may be a consequence of the introduction of more robust and risky play opportunities and the encouragement of schools to see the value of rough and tumble play. Thus, the finding may represent increased resilience and engagement in physical play, rather than an increase in undesirable behaviour.

Taken together, the findings of the present review are suggestive of an emerging field in need of more sensitive, valid and reliable ways to select and measure outcomes in social-emotional domains. Although it was encouraging to find in the quality assessment (Fig. 2) that most studies used previously validated instruments with good reliability, sensitivity to change within the study period was not considered. In fact, most of the studies included in our review did not set out to measure cognitive, social and emotional factors as primary outcomes, and therefore, the extant research has largely been designed with different interests in mind.

As discussed in the introduction, LPP has its roots in theory relating to design and creativity (Nicholson, 1972). Explicit reference to how psychological theory might link LPP to developmental outcomes in children was not always reflected in the chosen outcome or mediating measures for the included studies. For example, the null result for self-esteem in the Barton and colleagues study could be viewed as predictable given that SE is often conceptualised as a psychological ‘trait’ that is relatively stable over time (Robins & Trzesniewski, 2005). In future studies, the field would benefit if researchers specify exactly why or how an LPP intervention could be expected to shift outcomes such as SE over the course of the intervention and take steps to measure the mediating variables.

### Limitations of the Present Review

The present review has a number of limitations. Firstly, due to resource constraints we were unable to search systematically for grey literature, instead relying on professional networks to locate any relevant material. This means we have not assessed the extent to which publication bias could be affecting this field.

Another limitation is that the outcomes of interest are rather broadly specified. This was deliberate because one research aim was simply to get an overview of how outcome measures other than PA had been assessed. However, in future reviews, especially if researchers wish to conduct meta-analyses, tighter definitions of outcomes will be required.

Finally, this review does not contain a statistical meta-analysis of outcomes but has taken a thematic approach to evidence synthesis. This was the most appropriate approach given the heterogeneity of outcomes and the small number of studies; however, it is important that readers do not make inferences based on simple ‘vote counting’ (Thomas, O’Mara-Eves, Harden, & Newman, 2017), i.e. the practice of counting the number of statistically significant positive, neutral or negative differences in outcome between intervention and control groups. Vote counting would be problematic because it does not account for differences in study quality, sample size or effect size.

### Recommendations for Future Research

Notwithstanding the limitations mentioned above, we wish to highlight some key messages for future studies based on this overview of the extant research. Firstly, outcome measures should be carefully selected on the basis of their sensitivity to detect change as well as their construct validity. As discussed by the authors, the null findings in the Bundy and colleagues study demonstrate the importance of including measures which have a high enough ‘ceiling’ to allow detection of improvement in children who already have a high level of social competence.

Sampling is a related consideration as different populations may extract different benefits from interventions. Target populations for interventions were not explicitly reported by the included studies, and future studies could usefully address this issue. Groups at risk of low baseline cognitive, social and emotional development may potentially benefit more from playtime interventions (for example, those from deprived neighbourhoods). Therefore, researchers may wish to consider recruiting samples from at-risk groups in order to test for differential benefits. Alternatively researchers could aim to collect diverse samples and include suitable measures to allow investigation of the effects of factors such as SES.

A more rigorous approach to evaluating contextual influences is also recommended for future research. School playgrounds are heterogeneous environments, and it is possible that the characteristics of the playground could explain some of the variability in outcomes. As with any intervention based in a school, a good understanding of context is important to evaluating the reasons behind the observed results. Although contextual factors were often mentioned, they were mostly not systematically or quantitatively measured in our reviewed studies. Therefore, we here suggest some possible approaches which could be useful additions to future work.

A novel approach to understanding context was taken by Waters and Maynard in their study of child–teacher interactions in outdoor spaces (Waters & Maynard, 2010). Although this study is not directly concerned with a school-based LPP intervention, it is noteworthy for the methodology the researchers used to gain an insight into children’s perspectives. The researchers used microphones and video cameras to record the experiences of groups of children making outdoor excursions with their teachers. Data were then thematically coded, and patterns observed between the environmental characteristics and the initiations made by children in their conversations with teachers. The study found that around a third of child-initiated communications were around the discovery of naturally occurring Loose Parts in the environment. This example demonstrates the potential value of qualitative approaches in understanding the effects of LPP on children’s social behaviour as well, suggesting one way of assessing contextual influence.

Some studies have taken a quantitative approach to recording the characteristics of the playground environment and culture. For example, Chancellor and colleagues (Chancellor & Cevher-Kalburan, 2014) used a questionnaire methodology to investigate cross-cultural characteristics of playgrounds and schools. This technique could be used in the context of an LPP intervention pilot study in order to look for ways to optimise implementation, e.g. by having a record of the history of child involvement in design and planning of play spaces. Another advantage of collecting quantitative data on school and playground characteristics is that it allows for these features to be taken into account in later statistical analyses. For example, measures such as the number of pupils per square-metre, the amount of green-space per m^2^ could be included as controls in statistical models.

Our final recommendation concerns study design. Although RCTs are considered best practice in studies designed to test efficacy of interventions, our recommendation is that, given the limited quantity of research in the area, there is still space for small-scale, controlled or quasi-experimental pilot studies to explore issues of measurement, including validation, piloting and sensitivity. This would enable researchers to establish likely effectiveness and domains of interest before scaling up to RCTs involving multiple schools if indicated. As discussed above, different populations may benefit differently from interventions, so piloting across demographics may be informative.

## Conclusions

In conclusion, we note that the overall picture from the systematic review and narrative synthesis is of an emerging field at an exciting and crucial stage of development. Research of any kind concerning LPP is scarce and high-quality intervention research even more so. The questions arising from the theoretical perspectives are intriguing, and early indicators from the qualitative aspects of mixed-methods studies are promising. Nevertheless, the extent of the null results for social, cognitive and emotional outcomes in the two gold-standard RCT studies adds a cautionary note—the outcomes associated with LPP are by no means certain or established. More encouragingly, however, the overall picture is one of an absence of evidence, rather than robust evidence for an absence of positive effects.

## Footnotes

1 This study had follow-ups at 1 and 2 years post-intervention. Where results are null for both follow-ups, the OR is reported for the 2nd year only.
